# Toxin-Induced Methemoglobinemia With Kidney Injury and Hypoxic Brain Injury in a Case of Pesticide Poisoning: A Case Report

**DOI:** 10.7759/cureus.32516

**Published:** 2022-12-14

**Authors:** Kanishk K Adhit, Sharanya Menon, Sourya Acharya, Siddhaarth K

**Affiliations:** 1 Medicine, Jawaharlal Nehru Medical College, Datta Meghe Institute of Medical Sciences (Deemed to be University), Wardha, IND; 2 Medicine, Jawaharlal Nehru Medical College, Wardha, IND

**Keywords:** acute kidney injury, hypoxic encephalopathy, cyanosis, pesticide poisoning, methemoglobinemia

## Abstract

In many developing nations like India, the majority of the labor force comprises farmers. Therefore, there is a raised frequency of farmer suicides using pesticides. Toxin-induced methemoglobinemia is otherwise called toxic methemoglobinemia. It is a hematologic disorder attributed to exposure to toxic oxidizing agents and is most commonly seen in cases of poisoning. Methemoglobinemia is a condition in which there is an altered state of hemoglobin, resulting in reduced oxygen delivery to tissues. This case report represents a case of methemoglobinemia with acute kidney injury and hypoxic brain injury seen in a 23-year-old male patient. He was a farmer by occupation and was admitted due to ingestion of a pesticide named HUNT with suicidal intentions. He has had no previous history of psychiatric or neurologic disorders. The patient initially presented with a pulse rate of 110/min and room air saturation of 98% when he was brought to the casualty out patient department (OPD). Unfortunately, it worsened over the next 24 h, after which there was a sudden drop in SpO2 to 78% with oxygen support. Upon further examination and assessment, he was diagnosed with methemoglobinemia, leading to complications such as acute kidney failure and cerebral edema. He was then treated with hemodialysis, methylene blue, and ascorbic acid with viable improvement. This led to his complete recovery after eight days of treatment and support.

## Introduction

Methemoglobinemia is a potentially fatal condition. Through one study it was found that about one percent of the total number of patients with a history of self-inflicted pesticide poisoning had significant levels of methemoglobin (MetHb) out of which 85% of the patients had to be admitted. The mortality rate even after hospitalization in patients suffering from methemoglobinemia was 10% [1]. It occurs due to the reduced oxygen-carrying capacity of hemoglobin. It results due to the conversion of the reduced stage of ferrous iron to the oxidized state of ferric iron; heme in its ferric form will not be able to bind and carry oxygen, thereby leading to functional anemia [2]. Methemoglobinemia can be majorly divided into two types based on etiology, which are congenital type and acquired type. Congenital methemoglobinemia occurs due to genetic diseases, whereas, acquired methemoglobinemia occurs due to exposure to oxidizing drugs such as dapsone, benzocaine, and nitrates. Methemoglobinemia is a condition in which there is the presence of a greater concentration of MetHb in erythrocytes compared to the normal physiological concentration of 1%-2%. MetHb is dark blue in color, and clinical cyanosis occurs after concentrations exceed 15% [3]. Acquired methemoglobinemia mainly occurs due to exposure to certain chemicals or drugs in therapeutic doses or due to overdose. Neonates tend to be more susceptible to it [4]. The severity of signs and symptoms associated depends on the total amount of MetHb formed by the oxidation of hemoglobin. Patients are primarily asymptomatic in the initial stages. As the condition progresses, the classic findings are chocolate-colored blood, cyanosis, fatigue, lethargy, and dyspnea [5]. MetHb is generated by the oxidation of ferrous heme iron moieties to a ferric state. MetHb has a high affinity towards oxygen so that virtually no oxygen is delivered to the tissues. In this case, we describe a patient with a 42% MetHb level after ingestion of a pesticide. The pesticide was labeled and marketed as being safe to mammals and lacked details on toxicity and antidote anticipated when consumed in excess. 

Methemoglobinemia is typically diagnosed in clinical practice based on the patient's medical history and the symptoms or signs that the patient exhibit. This includes hypoxemia that is resistant to assisted oxygen supplementation and the probable existence of blood that is chocolate-colored. When MetHb is speciated from hemoglobin to determine the concentration and percent values of MetHb, the diagnosis of methemoglobinemia is validated by blood gas analysis from an artery or vein with co-oximetry [6]. Direct measures of SpO2 cannot be used to determine the severity of methemoglobinemia because light absorption in standard pulse oximetry is measured at wavelengths ranging only between 660 nm and 940 nm. MetHb absorbs light equally at both 660 nm wavelength and 940 nm wavelength; as a result, even if MetHb levels increase in the blood, oxygen saturation value would still remain the same due to equal absorbance [7]. Withdrawing the triggering agent and considering the use of the antidote methylene blue are both parts of treating methemoglobinemia. A mask that delivers high flow oxygen enhances the delivery of oxygen to tissues and encourages the spontaneous destruction of MetHb. This is through its interaction with the previously mentioned secondary pathway of MetHb reduction, where nicotinamide adenine dinucleotide phosphate (NADPH)-MetHb reductase uses NADPH from the glucose six phosphatase dehydrogenase (G6PD) dependent hexose monophosphate shunt to reduce methylene blue to leucomethylene blue. Methylene blue is generally known to act quickly and efficiently. Leucomethylene blue acts as an electron donor to transform MetHb into hemoglobin. Patients should be treated with methylene blue when MetHb exceeds 20%-30% in cases of acquired methemoglobinemia or at lower levels if the patient is symptomatic. Decisions about how to proceed with treatment must be based on the clinical picture rather than the results of laboratory tests. Methylene blue is given intravenously in doses of 0.1-0.2 mL/kg of a one percent solution or 1-2 mg/kg for 5 min [8]. If severe symptoms or levels continue to exceed the therapeutic threshold, the dose can be repeated in 30-60 min.

In scenarios where methylene blue treatment is unsuccessful or not recommended, other options can be resorted. Methylene blue would not be useful in patients having G6PD deficiency because G6PD helps in generating NADPH and methylene blue will only work in the presence of glycerol phosphate dehydrogenase (GLPD). Hence G6PD levels must be checked before giving methylene blue. This other options include ascorbic acid therapy, exchange transfusion, and hyperbaric oxygen therapy [9]. A higher dosage of ascorbic acid (vitamin C), up to 10 g/dose intravenously, can be used to treat MetHb [10]. It is unfortunately not as effective as anticipated and generally fails to provide the proper amount of care. Larger doses of ascorbic acid are associated with increased oxalate excretion in the urine when administered. When there is renal insufficiency, high doses of ascorbic acid may increase the risk of renal failure by producing hyperoxaluria [11]. 

## Case presentation

**Figure 1:**
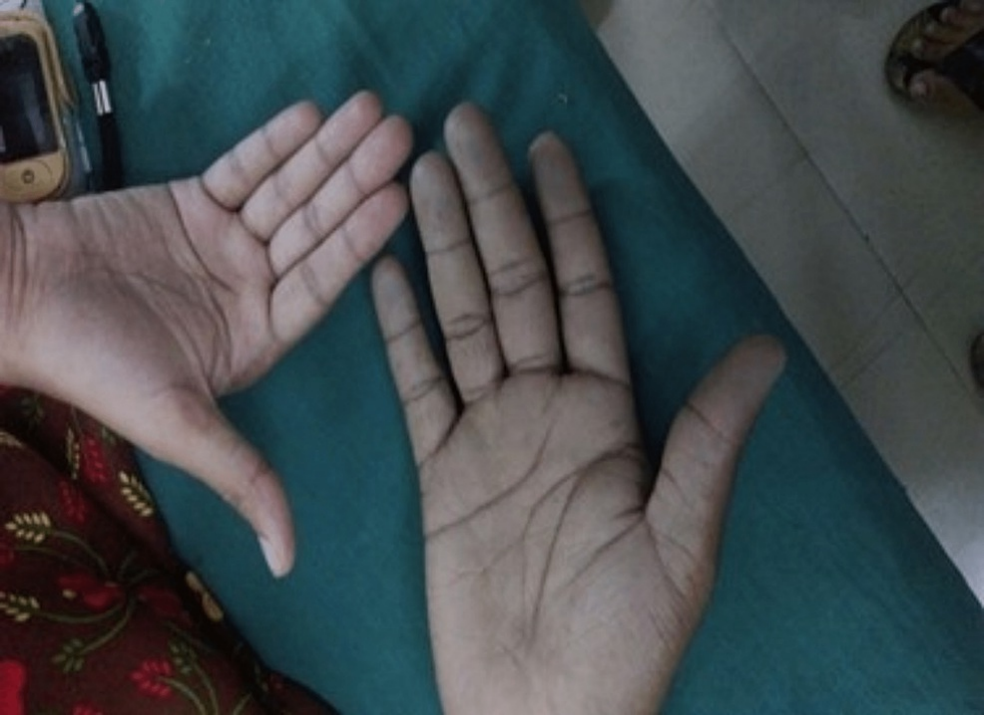
Cyanosis. Bluish discoloration of the skin seen in the patient hand (right side) in comparison with that of a normal person (left side).

**Figure 2:**
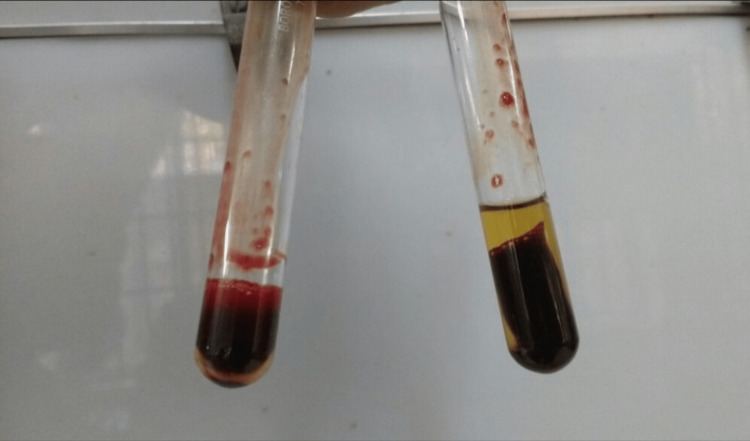
Comparison between the normal person and the patient's blood (showing chocolate brown colored blood). Chocolate brown colored blood on the right test tube due to methemoglobinemia

A 23-year-old male patient with no other medical illness allegedly consumed around 30 mL of a pesticide called ‘HUNT’ (contents were alkaloid suspended in natural oil, salts of unsaturated aliphatic carboxylic acid, emulsifiers, and media) with suicidal intent. He was found and brought to the hospital casualty outpatient department (OPD) in a drowsy state and got admitted with a pulse rate of 110/min, blood pressure of 110/70 mmHg, and saturation in room air by pulse oximetry was 98%. On day 2, the patient became awake and alert, but he developed headaches, diplopia, and cyanosis (Figure 1). His renal parameters urea and creatinine levels began to rise with a drop in the oxygen saturation (SpO2) by pulse oximetry. On day 1 the urea, creatinine, hemoglobin, and potassium values were 40 mg/dL, 1.2 mg/dL, 12 g/dL, 4 mmol/L respectively. On day 2 the urea levels were 68 mg/dL, creatinine was raised to 2.6 mg/dL, hemoglobin values dropped down to 7 g/dL, the renal parameters worsened on day 3 with creatinine levels increasing to 3.4 mg/dL and urea level also was increased to 75 mg/dL which indicated severe renal injury. Oxygen saturation was 78% when the patient was given 10 L of oxygen through a face mask. His arterial and venous blood sample was drawn and was noted to be chocolate brown in color (Figure 2), and arterial oxygen saturation (SaO2) by arterial blood gas (ABG) was 93%. These clinical findings were suggestive of methemoglobinemia. Methemoglobinemia was further confirmed by measuring its levels with pulse oximetry which was found to be 42%. Glucose 6 phosphate dehydrogenase (G6PD) levels were tested to rule out G6PD deficiency which could be a cause of methemoglobinemia and was found to be normal. G6PD helps in generating NADPH and methylene blue will work only in presence of G6PD. Hence G6PD levels must be checked before giving methylene blue. In the absence of G6PD, methylene blue will cause severe hemolysis.

**Figure 3:**
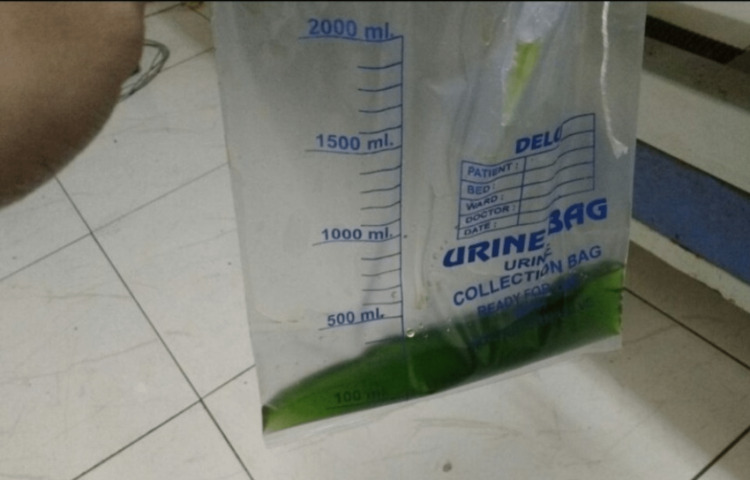
Urine color (maple syrup green color) after treatment with methylene blue.

As the patient had persistent headache and nausea, CT brain was done. CT brain showed features of cerebral edema. We proceeded with an MRI brain, which suggested features suggestive of hypoxic encephalopathy. His treatment included an IV injection of methylene blue at a dose of 1 mg/kg, a tablet of ascorbic acid 500 mg for five days, two cycles of hemodialysis, and two units of packed cell transfusion. Hemodialysis was done because the renal parameters dropped to significantly dangerous levels which could indicate acute renal injury. A red blood cell (RBC) transfusion was done as the patient had a drop in hemoglobin level on day 2 due to hemolysis. The pesticide composition was not known exactly to ascertain the exact cause of hemolysis. He was initially given ascorbic acid as it was readily available meanwhile G6PD deficiency was ruled out after which methylene blue was given. After treatment with methylene blue, the patient developed maple-green color urine (Figure 3). The patient gradually improved over a period of 8 days. He recovered with a saturation of 98% in room air. Subsequently, MetHb levels returned to one percent, and he had complete neurological recovery at the time of discharge. The renal parameters also came back to normal levels with urea values being 35 mg/dL and 1 mg/dL creatinine.

## Discussion

Methemoglobinemia is a condition where MetHb levels are higher than 2% in the blood. Methemoglobinemia must be suspected in patients with hypoxic symptoms and appearing cyanotic but having PaO2 levels high enough for hemoglobin to be fully saturable with oxygen. Low oxygen saturation on pulse oximetry and dark brown-colored blood could also be seen [12]. Acquired methemoglobinemia is usually caused by nitrite or other oxidant ingestion. Symptoms can be headache, nausea, vomiting, cyanosis, drowsiness, coma, and death. Parquet, indoxacarb, and aluminum phosphide are pesticides known to cause methemoglobinemia and acute renal failure [13]. In this particular case, the pesticide contains ingredients such as alkaloids suspended in natural oil. It also contains salts of unsaturated aliphatic carboxylic acid, emulsifiers, and media which were mentioned as safe for mammals. Alkaloids are naturally occurring chemical compounds that contain mostly basic nitrogen atoms. Hence, they can potentially cause methemoglobinemia [14]. MetHb is a type of hemoglobin in which there is the oxidation of the heme iron part which leads to the conversion of ferrous to ferric state. This affects oxygen affinity and thereby leads to hypoxia in the tissues. Methemoglobinemia occurs if there is a formation of MetHb at a higher rate when compared to cytochrome b5 reductive; there is improper unloading of oxygen in the base tissue levels also [15]. Most of these pesticides are marketed as safe for mammals and have no specific antidote as well. Through various studies, it has been found that methylene blue and exchange transfusion have proved to be successful in treating methemoglobinemia caused by poisoning. However, very high dosages and prolonged administration might lead to hypotension, chest pain, and hemolysis in patients suffering from G6PD deficiency because these individuals are susceptible to oxidative stress due to inadequate NADPH production [16]. In this case, the MetHb level was noticed to be 42%, with features of hemolysis, acute kidney injury, and signs of raised intracranial tension. Many toxins present in pesticides could have been the cause of injury to kidneys; hemolysis can be the main cause of acute kidney injury. He was treated with hemodialysis, methylene blue, vitamin C, and packed cell transfusion. After eight days, the patient's MetHb levels improved, it reached one percent saturation by pulse oximetry, and neurological symptoms also recovered completely. 

## Conclusions

Even though the compound was marketed as safe for mammals, it is advisable to be cautious about the composition of the products. This is because of the possibility that they may lead to various complications. Alkaloids could have caused methemoglobinemia in this patient. MetHb must be suspected and considered in any patient with cyanosis, partial pressure of oxygen (PaO2), oxygen saturation (SPO2) difference, and chocolate brown blood. The most common symptoms associated with methemoglobinemia are cyanosis, pallor, weakness, headache, seizures, and central nervous system depression, which might ultimately lead to coma and death when untreated. The most effective treatment for methemoglobinemia is administering methylene blue IV. MetHb and its complications can be managed with a high degree of suspicion and prompt treatment.

Farmer suicides in India amount to a significant percentage of total suicides in the country. Using pesticides is one of the most common means to attempt suicide among farmers. The majority of the patients with a history of pesticide poisoning associated with methemoglobinemia had to be hospitalized because of their severity and the mortality associated with such poisoning-induced methemoglobinemia was significantly higher indicating the importance of early identification and prompt treatment. In order to reduce the incidence and frequency of such pesticide poisonings, the Indian government should place stringent rules on the manufacturing and sale of pesticides. Vendors should also raise awareness among the customers regarding its safety. National and state-level regulations should be framed for the chemicals used to manufacture pesticides. A ban on highly hazardous pesticides from agricultural use should be considered. The government should spread awareness regarding the danger of ingesting harmful chemical agents in the form of pesticides among the people. This could be through newspapers and television. Special healthcare centers could be started in villages to help farmers cope with the psychological stress, and loan waivers could be offered by the government when in need.

With the advent of rapid development in the field of agriculture, there is the large-scale use of pesticides by farmers to increase yield. Along with it comes the increased danger of using pesticides as a means for suicide as it is easily available in their households. So, through this case report, we would like to ponder upon the importance and readiness of the primary health care centers in managing cases of poisoning as by effective and fast treatment the chances of mortality due to pesticide poisoning can be significantly reduced.
